# Iodine Nutritional Status and Thyroid Autoimmunity in Chinese Children and Adolescents Aged 6–17 Years

**DOI:** 10.3390/nu16213720

**Published:** 2024-10-30

**Authors:** Xueqing Li, Jiafeng Zhang, Hao Ding, Pengcheng Tu, Lizhi Wu, Mingluan Xing, Huixia Niu, Zhe Mo, Zhijian Chen

**Affiliations:** 1 Zhejiang Provincial Center for Disease Control and Prevention, Hangzhou 310051, China; xqli@cdc.zj.cn (X.L.); pchtu@cdc.zj.cn (P.T.); lzhwu@cdc.zj.cn (L.W.); mlxing@cdc.zj.cn (M.X.); 2111101061@nbu.edu.cn (H.N.); zhmo@cdc.zj.cn (Z.M.); 2 Hangzhou Institute for Food and Drug Control, Hangzhou 310051, China; zjfd0310@163.com; 3 Environmental Science Research & Design Institute of Zhejiang Province, Hangzhou 310051, China; asd198558@icloud.com

**Keywords:** iodine nutritional status, thyroid autoimmunity, thyroid autoantibodies, thyroid disorders

## Abstract

**Background:** Thyroid autoimmunity (TAI), marked by thyroid peroxidase antibodies (TPOAb) and thyroglobulin antibodies (TgAb), affects over 10% of the general population, with children and adolescents experiencing significant impacts on growth and quality of life despite lower prevalence rates compared to adults. **Methods:** In the context of over 20 years of universal salt iodization (USI) in China, this study investigated the relationship between iodine nutritional status and TAI in children and adolescents aged 6–17. **Results:** Our findings suggest that while iodine levels are generally sufficient (median urinary iodine concentration [UIC] was 205.2 µg/L), TAI remains a significant concern due to its potential impact on growth and development. TAI was significantly associated with age, sex, and urban–rural residency (*p* < 0.05). Positive TPOAb and TgAb were identified as risk factors for subclinical hypothyroidism (OR = 2.274, 95% CI: 1.171–1.916). Although some literature suggests that excessive iodine may exacerbate TAI and others propose iodine deficiency as a risk factor, this study did not find a significant overall association between iodine status and TAI. Notably, a low urinary iodine-to-creatinine ratio (UI/Cr) level was linked to an increased risk of TgAb positivity in males (OR = 3.470, 95% CI: 1.200–10.036). In individuals with negative thyroid antibodies, increased BMI (OR = 1.062, 95% CI: 1.032–1.093) and high UI/Cr levels (OR = 1.510, 95% CI: 1.175–1.941) were risk factors for subclinical hypothyroidism, whereas older age (OR = 0.710, 95% CI: 0.555–0.908 for the age 9–11 group; OR = 0.681, 95% CI = 0.484–0.959 for the age 12–17 group) and high UIC levels (OR = 0.739, 95% CI: 0.554–0.985) were associated with reduced risk. No significant associations were observed in the thyroid antibody-positive group. **Conclusions:** These results highlight the importance of considering individual TAI status when devising iodine supplementation policies.

## 1. Introduction

Autoimmune thyroid disease (AITD) is the most prevalent organ-specific autoimmune disorder, primarily encompassing Graves’ disease and Hashimoto’s thyroiditis [1]. Clinically, AITD is characterized by lymphocytic infiltration of the thyroid gland and the production of thyroid-specific antibodies, particularly thyroid peroxidase antibodies (TPOAb) and thyroglobulin antibodies (TgAb), which are well-recognized as pivotal markers of AITD [2]. Individuals testing positive for thyroid antibodies are generally considered to have thyroid autoimmunity (TAI), with a prevalence exceeding 10% in most populations [3]. Although the prevalence of thyroid antibody positivity in children and adolescents is lower than that in adults, these age groups are in critical developmental stages. Thyroid autoimmunity can adversely affect their learning abilities and quality of life to varying degrees and even impair their growth and development. Therefore, a comprehensive investigation of TAI in children and adolescents is essential for the early identification, diagnosis, and prevention of AITD.

TAI is typically the result of complex interactions between genetic susceptibility and environmental factors [4], with iodine intake potentially being the most significant environmental determinant [5]. Iodine is a crucial element for thyroid hormone synthesis, and both insufficient and excessive iodine intake can precipitate thyroid disorders. Historically, iodine deficiency has been considered a major public health concern. As one of the countries severely affected by iodine deficiency disorders (IDDs), China implemented a universal salt iodization (USI) program in 1995, successfully eliminating IDDs as a public health issue [6]. However, in recent years, the prevalence and distribution of thyroid disorders have increased, which has been partly attributed to excessive iodine intake, raising public concerns about iodine consumption, especially among urban coastal populations [7]. Consequently, academic research has shifted focus from iodine deficiency to the impact of excessive iodine intake on TAI [8]. Nevertheless, the relationship between iodine intake and TAI remains inconclusive, with inconsistent findings predominantly concentrated in adult populations and limited research involving children and adolescents. Thus, further investigation into the association between iodine nutritional status and TAI in these younger populations is of significant importance.

In addition to iodine intake, TAI is influenced by numerous other factors. In adult populations, females have been extensively studied and identified as the primary group affected by TAI, which may be related to hormonal fluctuations, inflammatory immune responses, and genetic differences [9,10,11]. Age is also a critical factor, as immune function and metabolic status undergo various changes over time, which may directly or indirectly influence TAI risk. Studies have shown a positive correlation between the levels of TPOAb and TgAb and age in iodine-sufficient regions, indicating an increase in antibody levels with advancing age [12]. This observation is consistent with findings from studies conducted in France [13] and Turkey [14], further affirming the significant role of age and gender in TAI. Mechanistic studies have suggested that leptin, sex hormone levels, and differential gene expression on the X chromosome are key factors underlying this age and gender distribution [15]. Furthermore, disparities between urban and rural areas may also impact TAI. Economic development and improved living standards have led to substantial changes in dietary habits, lifestyle, and environmental factors between urban and rural regions, potentially affecting TAI risk.

In the context of over 20 years of USI in China, we hypothesized that Chinese children and adolescents aged 6–17 years have a complex relationship between iodine nutritional status and TAI, one that is also affected by various factors such as age, gender, and urban-rural differences. Thus, the present study aimed to comprehensively assess the relationship between iodine nutritional status and TAI among children and adolescents in this age group, with a particular focus on the roles of age, gender, and urban–rural differences. Through this investigation, we hope to provide critical insights for the formulation of more scientifically grounded and reasonable iodine intake strategies by the government, ultimately safeguarding the thyroid health of Chinese children and adolescents.

## 2. Materials and Methods

This study was conducted in Chinese children and adolescents aged 6–17 years, focusing on their iodine nutritional status, while taking into the roles of age, gender, urban-rural differences, and body mass index (BMI). By comparing the rate of thyroid antibody positivity and the prevalence of thyroid disease, the relationship between iodine nutritional status and thyroid autoimmunity was explored.

### 2.1. Study Population

**Figure 1:**
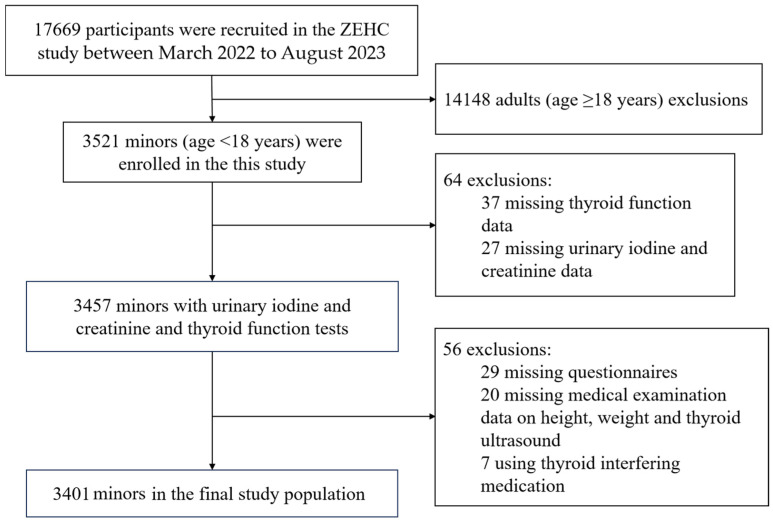
Flowchart of the selection of the study population.

The study population was selected from children and adolescents in the baseline population of the Zhejiang Environmental Health Cohort (ZEHC), an ongoing cohort designed by our team to investigate the associations between the environment and health in Zhejiang Province, China. From March 2022 to August 2023, 17,669 participants were recruited from four representative cities in Zhejiang: Huzhou (north), Jinhua (central), Taizhou (east), and Lishu (south), based on geographic location and economic conditions. The inclusion criteria for ZEHC were as follows: (1) local permanent residents aged 6–69 years; (2) no newly diagnosed or ongoing cancer treatment; (3) no mental illness or cognitive impairment; and (4) no intake of any iodine-containing drugs or contrast agents in the previous three months. In our study, only children and adolescents were selected (age 6–17 years), so adults (age ≥  18 years) were excluded (n = 14,148). Additionally, those without thyroid function data (n = 37), urine data (n = 27), questionnaires (n = 29), and medical examination (n = 20), as well as those using thyroid-interfering medication (n = 7), were also excluded. Finally, 3401 children and adolescents (age 6–17 years) were included in the analysis. A participant survey flowchart is shown in Figure 1.

All participants and their legal guardians signed the informed consent form at the baseline survey. The ZEHC was approved by the Zhejiang Provincial Center for Disease Control and Prevention.

### 2.2. Data Collection

All participants received standardized questionnaires and underwent medical examination by professional technicians, who had been trained and evaluated. The demographic characteristics included age, sex, height, body mass, and location. Height and body mass were measured using uniform equipment. BMI was calculated as body mass (kg) divided by height squared (m^2^). The medical examination included anthropometric measurements, routine examinations, and imaging tests. Among them, thyroid ultrasound was performed using a MicroMaxx portable color doppler ultrasound diagnostic apparatus (FUJIFILM SonoSite, Inc., Washington, DC, USA), with a transducer probe frequency of 7.5 MHz. The thyroid ultrasound examination was performed by one registered physician with an ultrasound professional certificate issued by the Ministry of Health of China and one assistant.

On the day of the medical examination, fasting blood and morning urine samples were collected at the local health clinic. Serum samples were centrifuged within 2 h of collection to detect thyroid function. To avoid iodine contamination during blood collection, alcohol disinfection was used instead of iodophor disinfection. Urine samples were collected in sterile urine cups to detect urinary iodine concentration (UIC) and urinary creatinine concentrations. Following the completion of the survey and specimen collection, all specimens were transported on the same day via a cold chain system to the central laboratory in Hangzhou, China, for immediate centralized testing.

### 2.3. Laboratory Measurements

We measured serum thyroid stimulating hormone (TSH), free triiodothyronine (FT3), free thyroxine (FT4), TgAb, and TPOAb using electrochemiluminescence immunoassays with a Cobas e601 analyzer (Roche Diagnostics GmbH, Mannheim, Germany), along with the appropriate calibration materials, reagents, and quality controls. Before, during, and after testing, the quality control procedure was followed as the manufacturer’s instructions. Control samples (PreciControl Universal and PreciControl ThyroAB, Roche, Germany) had the coefficient of variation (CV): TSH 2.75–5.57%, FT3 3.44–5.26%, FT4 2.26–4.23%, TgAb 3.83–6.63%, and TPOAb 5.41–8.33%. According to the test kit manufacturers, the reference ranges for TSH, FT4, TgAb, and TPOAb were 0.27–4.2 µIU/mL, 12–22 pmol/L, <115 IU/mL, and <34 IU/mL. Referencing Professor Teng’s research [16] and incorporating the reference range of our research laboratory, subclinical hypothyroidism was defined as having TSH > 4.2 mIU/L and FT4 within 12–22 pmol/L. Isolated positive TgAb (i-TgAb) was defined as having TgAb ≥ 115 IU/mL and TPOAb < 34 IU/mL. Isolated positive TPOAb (i-TPOAb) was defined as having TPOAb ≥ 34 IU/mL and TgAb < 115 IU/mL. Double-positive TgAb and TPOAb (d-Ab) were characterized by TgAb ≥ 115 IU/mL and TPOAb ≥ 34 IU/mL.

UIC was measured by inductively coupled plasma mass spectrometry (ICP-MS; ICAPR02041, Thermo Fisher Scientific, Bremen, Germany, GmbH). The CV of quality control samples (standard lyophilized human serum reference material, Trace Elements Urine L-1 RUO, Seronorm, Billingstad, Norway) was 3.17–4.24%. Urinary creatinine was measured with the automatic biochemical analyzer (Beckman AU480, Beckman Instruments, Brea, CA, USA) using the creatinine oxidase method. The CV of quality control samples (assayed urine chemistry control, AU2353, Randox Laboratories, Crumlin, UK) was 1.58–2.26%. The World Health Organization (WHO) recommended iodine nutrition assessment criteria were as follows: UIC < 100 µg/L as insufficient iodine intake, UIC 100–199 µg/L as adequate iodine intake, UIC 200–299 µg/L as more than adequate iodine intake, and UIC > 300 µg/L as excessive iodine intake.

### 2.4. Statistical Analyses

SPSS (version 26, NCSS Statistical Software) and Microsoft Excel (Win11 2021) were used for data processing and statistical analyses. The Kolmogorov–Smirnov method was adopted to test the normality of continuous variable distributions. Non-normally distributed data were expressed as the median with the interquartile range (IQR: 25th–75th percentiles) and analyzed using the Mann–Whitney U test or Kruskal–Wallis test for comparison of two or more group comparisons. Categorical variables were represented by counts and percentages, with the chi-squared (χ^2^) test or Fisher’s exact test employed for comparisons between groups. Logistic regression models were used to identify the associated factors of positive thyroid antibodies and evaluate the association of positive thyroid antibody and thyroid dysfunction with iodine nutritional status. UIC was stratified according to WHO criteria for assessing iodine status in the general population, while urinary iodine-to-creatinine ratio (UI/Cr) was stratified based on quartiles. The results were expressed with odds ratios (ORs) and 95% confidence intervals (CIs). All tests were two-tailed cutoffs, and significance was set at a 0.05 level (*p* < 0.05).

## 3. Results

### 3.1. Participant Characteristics

Participant characteristics are shown in Table 1. Males (52.1%) had higher BMI, FT3, FT4, and UIC, while females (47.9%) had higher positive TPOAb, positive TgAb, positive thyroid antibody, and thyroid nodule prevalence.

### 3.2. Prevalence of Positive Thyroid Antibodies

Table 2 shows positive thyroid antibody prevalence for each age, sex, region, and iodine intake group. There were differences in the prevalence of i-TgAb (*p* = 0.003) and d-Ab (*p* = 0.015) among age groups, with higher prevalence in age 9–11 and 12–17 years than in 6–8 years. The same age trend was observed for i-TgAb prevalence among females (*p* = 0.025). Among females, the prevalence of i-TgAb in urban areas was 4.9%; in contrast, the prevalence of i-TgAb in rural areas was 2.7%. Therefore, the prevalence of i-TgAb was higher in urban than in rural areas. Consistently, the prevalence of i-TPOAb was also higher in urban (3.8%) than in rural (2.1%) areas. Differences in positive thyroid antibody prevalence between iodine nutrient levels were not statistically significant (*p* > 0.05).

### 3.3. Thyroid Antibodies, Iodine Nutritional Status, and Associated Factors

Logistic regression analysis (Table 3) showed that the prevalence of positive i-TgAb and d-Ab significantly associated with increasing age (*p* < 0.05), with the same results obtained in females. Females who live in rural areas were inversely associated with positive antibodies (OR = 0.447, 95% CI: 0.249–0.800 for i-TgAb; OR = 0.445, 95% CI: 0.229–0.863 for i-TPOAb; OR = 0.559, 95% CI: 0.340–0.918 for d-Ab). However, no association was found between different UIC levels and thyroid antibodies. After correction for creatinine, males with low UI/Cr level (<111.5 µg/g) were at a higher risk of positive i-TgAb (OR = 3.470, 95% CI: 1.200–10.036).

### 3.4. Subclinical Hypothyroidism, Thyroid Nodules, Iodine Nutritional Status, and Associated Factors

**Figure 2:**
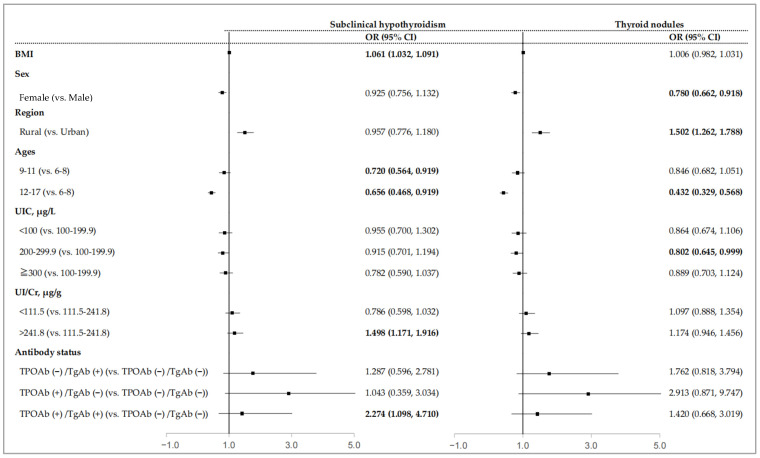
Association of subclinical hypothyroidism and thyroid nodules with iodine nutritional status and associated factors. Data are reported as odds ratios (confidence intervals). Bold remarks indicate statistically significant differences. Abbreviations: OR: odds ratio; 95% CI: 95% confidence interval; BMI, body mass index; UIC, urinary iodine concentration; UI/Cr, urinary iodine-to-creatinine ratio; TgAb, thyroglobulin antibody; TPOAb, thyroid peroxidase antibody.

As shown in Figure 2, females had a lower risk of thyroid nodules (OR = 0.780, 95% CI: 0.662–0.918), while participants living in rural areas had a higher risk of thyroid nodules (OR = 1.502, 95% CI: 1.262–1.788). BMI was positively associated with the prevalence of subclinical hypothyroidism (OR = 1.061, 95% CI: 1.032–1.091). Compared with the age 6–8 group, the risk of subclinical hypothyroidism was lower in the age 9–11 and 12–17 groups (OR = 0.720, 95% CI: 0.564–0.919 for the age 9–11 group; OR = 0.656, 95% CI = 0.468–0.919 for the age 12–17 group), and the risk of thyroid nodules was lower in the age 12–17 group (OR = 0.432, 95% CI: 0.329–0.568). More than adequate iodine intake (UIC 200–299.9 µg/L) was inversely associated with thyroid nodules (OR = 0.802, 95% CI: 0.645–0.999). Participants with high UI/Cr levels (>241.8 µg/g) were at a higher risk of subclinical hypothyroidism (OR = 1.498, 95% CI: 1.171–1.916). Compared with the negative antibody group, the positive d-Ab group had a higher risk of subclinical hypothyroidism (OR = 2.274, 95% CI: 1.171–1.916).

Further, the participants were classified into thyroid-antibody-positive and -negative groups (Table 4 and Table 5). In the negative antibody group, the associations of age, BMI, region, sex, and UI/Cr levels with subclinical hypothyroidism and thyroid nodules were consistent with the results obtained from all participants. Differently, excessive iodine intake (UIC ≥ 300 µg/L) was inversely associated with subclinical hypothyroidism (OR = 0.739, 95% CI: 0.554–0.985), and more than adequate iodine intake (UIC 200–299.9 µg/L) was not associated with thyroid nodules (*p* = 0.055). However, in the positive antibody group, no associations were found between subclinical hypothyroidism and thyroid nodules with associated factors.

## 4. Discussion

In the context of over two decades of the implementation of the USI strategy in China, investigating the relationship between iodine nutritional status and TAI has significant public health implications. Current research on TAI remains limited, predominantly focusing on adult populations, with relatively few studies involving children and adolescents. Thus, enhancing research on TAI in these younger populations is crucial for early identification, diagnosis, and prevention of TAI. In the present study, we found that the UIC and UI/Cr levels among Chinese children and adolescents aged 6–17 years were 205.2 µg/L (127.0, 313.6) and 167.8 µg/g (111.5, 241.8), respectively, indicating a state of adequate iodine nutrition. The prevalence rates of i-TPOAb, i-TgAb, d-Ab, subclinical hypothyroidism, and thyroid nodules were 2.1%, 2.7%, 3.6%, 13.8%, and 23.5%, respectively. A study conducted in Spain reported a TAI prevalence of 3.7% among children and adolescents aged 1–16 years [17], and data from the Korea National Health and Nutrition Examination Survey (KNHANES) indicated a TPOAb positivity rate of 2.3% among individuals aged 10–19 years [18]. Therefore, the prevalence of TAI among Chinese children and adolescents is comparable to these reported studies. Although the prevalence of TAI in children and adolescents is lower than that in adults (approximately 10–13%), these age groups are at critical developmental stages, and TAI can negatively affect their learning abilities, quality of life, and even growth and development to varying degrees. Moreover, our study identified significant associations between age, gender, and urban–rural residence and TAI, with dual positivity for TPOAb and TgAb being a risk factor for subclinical hypothyroidism.

For a long time, iodine has been considered an environmental determinant of thyroid dysfunction; however, its relationship with TAI remains controversial. Some studies suggest that excessive iodine intake may trigger or exacerbate TAI. Pescopagano et al. reported that, 15 years after the implementation of the USI strategy, the median urinary iodine level increased from 55 µg/L to 98 µg/L, and the thyroid antibody positivity rate rose from 12.6% to 19.5% [19]. In an autoimmune-prone NOD.H-2h4 mouse model, it was demonstrated that high-iodine diets induced spontaneous thyroiditis and the development of thyroid autoantibodies, predominantly TgAb [20]. In contrast, a national cross-sectional survey in mainland China found an inverse relationship between iodine intake and thyroid antibody levels, with the highest TPOAb and TgAb positivity rates observed in iodine-deficient groups, suggesting that iodine deficiency is a risk factor for TAI [21]. Another study conducted two decades after the implementation of the USI strategy in China indicated that excessive iodine intake may be a protective factor against TPOAb positivity [22]. However, some studies have found no association between increased iodine intake and TAI [8,23,24,25]. A large cross-sectional study by Zimmermann et al. showed that neither iodine deficiency nor excessive iodine intake in children increased the risk of TAI [26]. Our study found no significant association between overall iodine nutritional status and TAI, except that males with lower UI/Cr levels had an increased risk of i-TgAb positivity. Currently, there are discrepancies regarding the relationship between iodine and thyroid autoimmunity, which may be closely related to another key element—selenium. Selenium is also an essential trace element for the synthesis of thyroid hormones, and the thyroid gland contains the most selenium of all organs [27]. Selenium possesses strong antioxidant properties, aiding in the scavenging of free radicals and exhibiting a certain degree of antagonism against damage caused by both high and low iodine levels. Additionally, it provides protective effects against injuries induced by autoimmune thyroid diseases. Studies have found that both low and high iodine statuses are associated with autoimmune thyroid diseases, with a common characteristic being a low selenium status [28]. Our study did not find a significant overall association between iodine status and TAI, which may be due to adequate selenium intake resulting from improved living standards, thereby exerting a protective effect on the body. Therefore, future research should include selenium as an important factor.

Although no direct association between iodine nutritional status and TAI was observed in our study, our previous research identified a relationship between iodine nutritional status and subclinical hypothyroidism and thyroid nodules. Consequently, we further explored whether TAI mediates this relationship and found that children and adolescents with dual positivity for TPOAb and TgAb had a higher risk of subclinical hypothyroidism. When stratifying children and adolescents into antibody-positive and antibody-negative groups, contrasting results emerged regarding the association between iodine nutritional status and thyroid diseases. In the antibody-negative group, we found that increased BMI and high UI/Cr levels were risk factors for subclinical hypothyroidism, while individuals aged 9–17 years and those with high UIC levels had a lower risk of subclinical hypothyroidism. Notably, different results were obtained using different iodine assessment indicators (UI/Cr and UIC), potentially due to the influence of water and dietary intake on UIC, which may not accurately reflect individual iodine nutritional status. In contrast, creatinine can effectively correct the impact of urine volume on urinary iodine concentration [29]. Thus, increasing studies have utilized UI/Cr to assess iodine nutritional status [30,31,32]. A study in mainland China found that the risk of subclinical hypothyroidism was higher in iodine-sufficient and excessive iodine areas compared to iodine-deficient areas [33]. Additionally, animal studies have demonstrated that prolonged high-iodine intake can inhibit pituitary type 2 deiodinase activity, leading to reduced conversion of T4 to T3 and increased TSH production [34]. Therefore, our findings suggest that UI/Cr may be more suitable for assessing individual iodine nutritional status. However, in the antibody-positive group, no significant associations were observed between iodine nutritional status and other factors with subclinical hypothyroidism or thyroid nodules. This indicates that individuals with different TAI statuses may have varying iodine requirements and thyroid disease risks, highlighting the critical role of TAI in the relationship between iodine nutritional status and thyroid diseases and underscoring the need to consider individual TAI status in the formulation of iodine supplementation strategies and precise prevention and control of thyroid diseases. When treating thyroid diseases, it is important to consider the patient’s specific conditions, such as their TAI status and iodine nutrition levels, in order to devise a more precise treatment plan and improve treatment outcomes.

In addition to iodine, numerous other factors play significant roles in the pathogenesis of TAI. Studies have found that thyroid autoantibodies are more prevalent in females, with rates at least 10 times higher than in males [35]. This gender disparity may be attributed to factors such as the X chromosome, pregnancy, and hormones [36]. Research has shown that the gender difference in TAI manifests during puberty, when leptin and estrogen levels simultaneously increase. Both leptin and estrogen are effective modulators of immune responses [37,38]. Leptin has also been implicated in contributing to the gender disparity in disease prevalence [39]. Our study further confirmed that the prevalence of thyroid autoantibody positivity in female children and adolescents was 2.6 times higher than in their male counterparts. In the female i-TgAb and d-Ab groups, the risk of TAI was significantly higher in adolescents aged 9–17 years compared to children aged 6–8 years. In the male i-TgAb group, the ORs were also large for both the 9–11 (OR = 7.421) and 12–17 (OR = 4.058) aged groups, with no statistical significance however, probably due to relatively small positive samples. In reality, the prevalence of i-TgAb positivity is quite low, which is consistent with previous reports [21]. With advancing age, the immune function and metabolic status of the human body undergo various changes, which may directly or indirectly influence the risk of TAI. Besides age and gender, our study also found that children and adolescents living in urban areas had a higher risk of TAI, potentially related to differences in dietary habits, lifestyle, and environmental factors between urban and rural areas, which warrants further investigation. These findings can assist clinicians in optimizing screening and diagnostic strategies by conducting more targeted tests on high-risk populations (e.g., females, adolescents, and urban residents), thereby enabling early detection of thyroid issues and enhancing the accuracy of diagnoses.

This study is the first to report the prevalence of TAI among Chinese children and adolescents aged 6–17 years while comprehensively exploring the relationship between iodine nutritional status and TAI, incorporating factors such as age, gender, and urban–rural differences. It also uniquely highlights the disparities between urban and rural areas, which require further research for detailed elucidation. The limitations of this study include its baseline cross-sectional design, which precludes establishing causal relationships between iodine nutritional status and TAI. However, as cohort studies are conducted in the future, we will accumulate more longitudinal data to refine the analysis. Additionally, there is a high possibility of iodine–gene interactions in the development of TAI. For instance, in individuals with susceptible genetic backgrounds, the relationship between iodine levels and TAI risk remains underexplored and warrants further investigation.

## 5. Conclusions

In summary, children and adolescents aged 6–17 years in Zhejiang, China, exhibit adequate iodine nutritional status, with thyroid autoantibody positivity rates lower than those in adult populations. Our study found no significant association between iodine nutritional status and TAI, but identified age, gender, and urban–rural residence as important influencing factors for TAI. Given the close relationship between TAI and thyroid diseases, individuals with dual positivity for TPOAb and TgAb are at a higher risk of subclinical hypothyroidism. Our findings also suggest that when monitoring iodine supplementation for various types of thyroid diseases, individual TAI status should be carefully considered.

## Figures and Tables

**Table 1:** Characteristics of study subjects and the prevalence of thyroid-related parameters in males and females.

Variable	Total	Male	Female	*p*-Value
N (%)	3401 (100.0)	1772 (52.1)	1629 (47.9)	
Age, years, median (IQR)	10.0 (9.0, 11.0)	10.0 (9.0, 11.0)	10.0 (9.0, 11.0)	0.979
BMI, kg/m^2^, median (IQR)	17.1 (15.5, 20.0)	17.5 (15.7, 20.8)	16.7 (15.3, 19.1)	**<0.001**
Region				
Urban	2048 (60.2)	1051 (59.3)	997 (61.2)	0.262
Rural	1353 (39.8)	721 (40.7)	632 (38.8)	
Thyroid function indexes				
FT3, pmol/L, median (IQR)	6.8 (6.2, 7.4)	6.8 (6.3, 7.5)	6.7 (6.1, 7.3)	**0.004**
FT4, pmol/L, median (IQR)	18.0 (16.4, 19.6)	18.2 (16.5, 19.7)	17.8 (16.2, 19.4)	**<0.001**
TSH, µIU/mL, median (IQR)	2.6 (1.9, 3.5)	2.6 (1.9, 3.6)	2.5 (1.8, 3.4)	0.110
Tg, ng/mL, median (IQR)	12.2 (8.2,18.2)	12.1 (8.4, 17.7)	12.4 (7.9, 18.7)	0.528
Iodine nutrient levels				
UIC, µg/L, median (IQR)	205.2 (127.0, 313.6)	216.0 (134.0, 323.0)	195.7 (118.0, 307.0)	**<0.001**
UI/Cr, µg/g, median (IQR)	167.8 (111.5, 241.8)	166.7 (112.1, 239.9)	168.3 (111.3, 243.3)	0.619
Thyroid disorders				
Positive TPOAb, n (%)	71 (2.1)	20 (1.1)	51 (3.1)	**<0.001**
Positive TgAb, n (%)	92 (2.7)	26 (1.5)	66 (4.1)	**<0.001**
Positive thyroid antibodies, n (%)	121 (3.6)	36 (2.0)	85 (5.2)	**<0.001**
Subclinical hypothyroidism, n (%)	469 (13.8)	256 (14.4)	213 (13.1)	0.247
Thyroid nodules, n (%)	798 (23.5)	380 (21.4)	418 (25.7)	**0.004**

*p*-values are for trends between males and females. Bold remarks indicate statistically significant differences. Abbreviations: BMI, body mass index; IQR, interquartile range; TSH, thyroid-stimulating hormone; FT3, free triiodothyronine; FT4, free thyroxine; Tg, thyroglobulin; UIC, urinary iodine concentration; UI/Cr, urinary iodine-to-creatinine ratio; TgAb, thyroglobulin antibody; TPOAb, thyroid peroxidase antibody.

**Table 2:** Prevalence of positive thyroid antibodies stratified by sex.

	Total				Male				Female			
	N	i-TgAb, n (%)	i-TPOAb, n (%)	d-Ab, n (%)	N	i-TgAb, n (%)	i-TPOAb, n (%)	d-Ab, n (%)	N	i-TgAb, n (%)	i-TPOAb, n (%)	d-Ab, n (%)
Ages												
6–8	759	7 (0.9)	11 (1.4)	14 (1.8)	388	1 (0.3)	2 (0.5)	3 (0.8)	371	6 (1.6)	9 (2.4)	11 (3.0)
9–11	1955	64 (3.3) ^#^	42 (2.1)	78 (4.0) ^#^	1023	20 (2.0)	13 (1.3)	24 (2.3)	932	44 (4.7) ^#^	29 (3.1)	54 (5.8)
12–17	687	21 (3.1) ^#^	18 (2.6)	29 (4.2) ^#^	361	5 (1.4)	5 (1.4)	9 (2.5)	326	16 (4.9) ^#^	13 (4.0)	20 (6.1)
χ^2^		11.890	2.501	8.438		5.627	2.014	3.982		7.345	1.398	4.987
*p*		**0.003**	0.286	**0.015**		0.060	0.365	0.137		**0.025**	0.497	0.083
Region												
Urban	2048	63 (3.1)	48 (2.3)	78 (3.8)	1051	14 (1.3)	10 (1.0)	18 (1.7)	997	49 (4.9)	38 (3.8)	60 (6.0)
Rural	1353	29 (2.1)	23 (1.7)	43 (3.2)	721	12 (1.7)	10 (1.4)	18 (2.5)	632	17 (2.7)	13 (2.1)	25 (4.0)
χ^2^		2.693	1.652	0.944		0.327	0.727	1.320		4.925	3.926	3.327
*p*		0.101	0.199	0.331		0.568	0.394	0.251		**0.026**	**0.048**	0.068
UIC, µg/L												
<100	548	17 (3.1)	13 (2.4)	21 (3.8)	232	2 (0.9)	4 (1.7)	5 (2.2)	316	15 (4.7)	9 (2.8)	16 (5.1)
100–199.9	1094	26 (2.4)	26 (2.4)	38 (3.5)	576	8 (1.4)	7 (1.2)	12 (2.1)	518	18 (3.5)	19 (3.7)	26 (5.0)
200–299.9	824	23 (2.8)	19 (2.3)	30 (3.6)	456	6 (1.3)	3 (0.7)	7 (1.5)	368	17 (4.6)	16 (4.3)	23 (6.3)
≥300	935	26 (2.8)	13 (1.4)	32 (3.4)	508	10 (2.0)	6 (1.2)	12 (2.4)	427	16 (3.7)	7 (1.6)	20 (4.7)
χ^2^		0.820	3.080	0.209		1.330	1.891	0.942		1.243	5.505	1.095
*p*		0.845	0.379	0.976		0.741	0.594	0.830		0.743	0.138	0.778
UI/Cr, µg/g												
<111.5 (Quartile 1)	850	28 (3.3)	23 (2.7)	37 (4.4)	439	10 (2.3)	6 (1.4)	13 (3.0)	411	18 (4.4)	17 (4.1)	24 (5.8)
111.5–241.8 (Quartile 2 + 3)	1700	40 (2.4)	37 (2.2)	55 (3.2)	892	8 (0.9)	12 (1.3)	15 (1.7)	808	32 (4.0)	25 (3.1)	40 (5.0)
>241.8 (Quartile 4)	851	24 (2.8)	11 (1.3)	29 (3.4)	441	8 (1.8)	2 (0.5)	8 (1.8)	410	16 (3.9)	9 (2.2)	21 (5.1)
χ^2^		1.964	4.287	2.137		4.370	2.890	2.560		0.154	2.557	0.445
*p*		0.374	0.117	0.343		0.112	0.236	0.278		0.926	0.278	0.800

^#^ Significance between two subgroups using the chi-squared test with a Bonferroni correction, as well as chi-squared test results compared with results obtained from age 6–8 years, *p* < 0.05. Bold remarks indicate statistically significant differences. Abbreviations: UIC, urinary iodine concentration; UI/Cr, urinary iodine-to-creatinine ratio; TgAb, thyroglobulin antibody; TPOAb, thyroid peroxidase antibody; d-Ab, double-positive thyroglobulin antibody and thyroid peroxidase antibody.

**Table 3:** Association of positive thyroid antibodies, iodine nutritional status, and associated factors.

	i-TgAb OR (95% CI)			i-TPOAb OR (95% CI)			d-Ab OR (95% CI)		
	Total	Male	Female	Total	Male	Female	Total	Male	Female
Ages									
6–8	1.000 (reference)								
9–11	**4.030** (1.817, 8.937) *	7.421 (0.973, 56.585)	**3.464** (1.436, 8.354) *	1.577 (0.793, 3.136)	2.108 (0.455, 9.761)	1.469 (0.670, 3.220)	**2.343** (1.302, 4.219) *	2.753 (0.804, 9.426)	**2.240** (1.135, 4.422) *
12–17	**4.298** (1.716, 10.763) *	4.058 (0.435, 37.808)	**4.586** (1.633, 12.883) *	2.058 (0.890, 4.759)	1.990 (0.341, 11.598)	2.178 (0.818, 5.796)	**2.644** (1.309, 5.341) *	2.420 (0.596, 9.832)	**2.755** (1.197, 6.344) *
Region									
Urban	1.000 (reference)								
Rural	**0.611** (0.386, 0.968) *	1.167 (0.522, 2.606)	**0.447** (0.249, 0.800) *	0.623 (0.370, 1.047)	1.299 (0.522, 3.230)	**0.445** (0.229, 0.863) *	0.738 (0.499, 1.092)	1.320 (0.668, 2.608)	**0.559** (0.340, 0.918) *
BMI	0.969 (0.910, 1.031)	0.990 (0.892, 1.099)	0.981 (0.904, 1.066)	0.977 (0.910, 1.048)	1.021 (0.912, 1.145)	0.981 (0.893, 1.078)	0.980 (0.929, 1.035)	1.012 (0.929, 1.103)	0.989 (0.920, 1.064)
UIC, µg/L									
<100	1.222 (0.651, 2.292)	0.515 (0.106, 2.498)	1.371 (0.673, 2.792)	0.947 (0.479, 1.874)	1.444 (0.409, 5.095)	0.737 (0.326, 1.667)	1.051 (0.606, 1.822)	0.989 (0.338, 2.891)	0.981 (0.513, 1.874)
100–199.9	1.000 (reference)								
200–299.9	1.214 (0.679, 2.169)	1.118 (0.374, 3.343)	1.343 (0.672, 2.684)	1.024 (0.556, 1.883)	0.563 (0.143, 2.216)	1.267 (0.631, 2.546)	1.080 (0.657, 1.776)	0.792 (0.304, 2.062)	1.277 (0.707, 2.308)
≥300	1.155 (0.621, 2.151)	2.033 (0.652, 6.333)	0.987 (0.457, 2.131)	0.691 (0.331, 1.443)	1.296 (0.391, 4.295)	0.502 (0.191, 1.319)	1.012 (0.591, 1.733)	1.473 (0.579, 3.747)	0.883 (0.450, 1.734)
UI/Cr, µg/g									
<111.5 (Quartile 1)	1.377 (0.806, 2.351)	**3.470** (1.200, 10.036) *	1.053 (0.556, 1.996)	1.144 (0.645, 2.028)	0.906 (0.303, 2.715)	1.349 (0.684, 2.661)	1.300 (0.819, 2.064)	1.802 (0.775, 4.188)	1.192 (0.679, 2.092)
111.5–241.8 (Quartile 2 + 3)	1.000 (reference)								
>241.8 (Quartile 4)	1.241 (0.713, 2.160)	1.708 (0.596, 4.891)	1.122 (0.576, 2.185)	0.671 (0.327, 1.378)	0.345 (0.072, 1.654)	0.886 (0.388, 2.022)	1.112 (0.679, 1.820)	1.055 (0.418, 2.661)	1.166 (0.646, 2.102)

Bold remarks indicate statistically significant differences. * *p* < 0.05. Abbreviations: OR: odds ratio; 95% CI: 95% confidence interval; BMI, body mass index; UIC, urinary iodine concentration; UI/Cr, urinary iodine-to-creatinine ratio; TgAb, thyroglobulin antibody; TPOAb, thyroid peroxidase antibody; d-Ab, double-positive thyroglobulin antibody and thyroid peroxidase antibody.

**Table 4:** Association of subclinical hypothyroidism, iodine nutritional status, and associated factors in participants with and without positive thyroid antibodies.

	Subclinical Hypothyroidism			
	TPOAb (−)/TgAb (−)		TPOAb (+) or TgAb (+)	
	OR (95% CI)	*p*	OR (95% CI)	*p*
BMI	**1.062 (1.032, 1.093)**	**<0.001**	1.033 (0.897, 1.190)	0.648
Ages				
6–8	Reference		Reference	
9–11	**0.710 (0.555, 0.908)**	**0.006**	1.141 (0.204, 6.392)	0.881
12–17	**0.681 (0.484, 0.959)**	**0.028**	0.355 (0.040, 3.171)	0.354
Sex				
Male				
Female	0.915 (0.745, 1.124)	0.398	1.591 (0.523, 4.840)	0.414
Region				
Urban				
Rural	0.915 (0.738, 1.134)	0.417	2.486 (0.782, 7.899)	0.123
UIC, µg/L				
<100	0.978 (0.714, 1.340)	0.891	0.707 (0.119, 4.202)	0.703
100–199.9				
200–299.9	0.893 (0.680, 1.172)	0.414	1.658 (0.432, 6.368)	0.461
≥300	**0.739 (0.554, 0.985)**	**0.039**	2.851 (0.628, 12.947)	0.175
UI/Cr, µg/g				
<111.5 (Quartile 1)	0.765 (0.578, 1.013)	0.061	1.136 (0.294, 4.397)	0.853
111.5–241.8 (Quartile 2 + Quartile 3)				
>241.8 (Quartile 4)	**1.510 (1.175, 1.941)**	**0.001**	1.207 (0.316, 4.615)	0.783

Bold remarks indicate statistically significant differences. Abbreviations: OR: odds ratio; 95% CI: 95% confidence interval; BMI, body mass index; UIC, urinary iodine concentration; UI/Cr, urinary iodine-to-creatinine ratio; TgAb, thyroglobulin antibody; TPOAb, thyroid peroxidase antibody.

**Table 5:** Association between thyroid nodules, iodine nutritional status, and associated factors in participants with and without positive thyroid antibodies.

	Thyroid Nodules			
	TPOAb (−)/TgAb (−)		TPOAb (+) or TgAb (+)	
	OR (95% CI)	*p*	OR (95% CI)	*p*
BMI	1.005 (0.981, 1.029)	0.707	1.086 (0.914, 1.291)	0.349
Ages				
6–8	Reference		Reference	
9–11	0.843 (0.677, 1.049)	0.126	0.947 (0.205, 4.376)	0.945
12–17	**0.428 (0.325, 0.564)**	**<0.001**	0.585 (0.087, 3.946)	0.582
Sex				
Male				
Female	**0.780 (0.662, 0.920)**	**0.003**	0.721 (0.207, 2.510)	0.607
Region				
Urban				
Rural	**1.512 (1.267, 1.804)**	**<0.001**	1.018 (0.305, 3.405)	0.976
UIC, µg/L				
<100	0.879 (0.683, 1.130)	0.314	0.413 (0.099, 1.731)	0.227
100–199.9				
200–299.9	0.805 (0.645, 1.005)	0.055	0.640 (0.167, 2.451)	0.515
≥300	0.878 (0.692, 1.113)	0.281	1.543 (0.297, 8.010)	0.606
UI/Cr, µg/g				
<111.5 (Quartile 1)	1.088 (0.878, 1.348)	0.441	1.345 (0.380, 4.756)	0.646
111.5–241.8 (Quartile 2 + Quartile 3)				
>241.8 (Quartile 4)	1.197 (0.962, 1.489)	0.107	0.506 (0.123, 2.076)	0.344

Bold remarks indicate statistically significant differences. Abbreviations: OR: odds ratio; 95% CI: 95% confidence interval; BMI, body mass index; UIC, urinary iodine concentration; UI/Cr, urinary iodine-to-creatinine ratio; TgAb, thyroglobulin antibody; TPOAb, thyroid peroxidase antibody.

## Data Availability

The data presented in this study are available on request from the corresponding author. The data are not publicly available due to them involving personal information.
